# Effects of the breed, sex and age on cellular content and growth factor release from equine pure-platelet rich plasma and pure-platelet rich gel

**DOI:** 10.1186/1746-6148-9-29

**Published:** 2013-02-12

**Authors:** Carlos E Giraldo, Catalina López, María E Álvarez, Ismael J Samudio, Marta Prades, Jorge U Carmona

**Affiliations:** 1 Grupo de Investigación Terapia Regenerativa, Departamento de Salud Animal, Universidad de Caldas, Manizales, Colombia; 2 Grupo de Terapia Celular y Molecular, Departamento de Nutrición y Bioquímica, Pontificia Universidad Javeriana, Bogotá, Colombia; 3 Departamento de Medicina y Cirugía Animal, Facultad de Veterinaria, Universidad Autónoma de Barcelona, Cerdanyola del Vallès, Spain

**Keywords:** Horse, Platelet concentrate, Transforming growth factor beta 1, Platelet derived growth factor isoform BB, Regenerative therapy

## Abstract

**Background:**

There is no information on the effects of the breed, gender and age on the cellular content and growth factor (GF) release from equine pure-platelet rich plasma (P-PRP) and pure-platelet rich gel (P-PRG). The objectives of this study were: 1) to compare the cellular composition of P-PRP with whole blood and platelet poor plasma (PPP); 2) to compare the concentration of transforming GF beta 1 (TGF-β_1_) and platelet derived GF isoform BB (PDGF-BB) between P-PRP treated with non-ionic detergent (P-PRP+NID), P-PRG (activated with calcium gluconate -CG-), PPP+NID, PPP gel (PPG), and plasma and; 3) to evaluate and to correlate the effect of the breed, gender and age on the cellular and GF concentration for each blood component. Forty adult horses, 20 Argentinean Creole Horses (ACH) and, 20 Colombian Creole Horses (CCH) were included. Data were analyzed by parametric (i.e.: t-test, one way ANOVA) and non parametric (Kruskal-Wallis test, Wilcoxon test) tests. Correlation analysis was also performed by using the Spearman and Pearson tests. A p ≤ 0.05 was set as significant for all tests. All the blood components were compared for platelet (PLT), leukocyte (WBC), TGF-β_1_ and PDGF-BB concentrations. The effect of the breed, gender and age on these variables was analyzed. A P ≤ 0.05 was accepted as significant for all the tests.

**Results:**

PLT counts were 1.8 and 0.6 times higher in P-PRP than in whole blood and PPP, respectively; WBC counts were 0.5 and 0.1 times lower in P-PRP, in comparison with whole blood and PPP, respectively. TGF-β_1_ and PDGF-BB concentrations were 2.3 and 262 times higher, respectively, in P-PRG than in plasma, and 0.59 and 0.48 times higher, respectively, in P-PRG than in PPG. P-PRG derived from CCH females or young horses presented significantly (P < 0.001) higher PDGF-BB concentrations than P-PRG derived from ACH males or older horses.

**Conclusions:**

Our results indicated that P-PRP obtained by a manual method was affected by intrinsic factors such as the breed, gender and age. Equine practitioners should be aware that cellular and GF release from P-PRP/P-PRG could change according with the intrinsic variables associated with a patient in particular**.**

## Background

The clinical use of platelet concentrates (PC) is currently a common biological therapy for musculoskeletal diseases [1-4] and wounds in horses [5]. PC intended for regenerative medicine are classified as: pure-platelet rich plasma (P-PRP), leukocyte-platelet rich plasma (L-PRP) and platelet rich fibrin (PRF). P-PRP and L-PRP are obtained in a liquid form by using anticoagulants. PRF is as a second generation PC that does not require anticoagulant for its elaboration [6].

Equine P-PRP displays slightly higher platelet counts (1.3-4 fold) and leukocyte (WBC) counts (0.5-2 fold) than whole blood. Equine L-PRP has increased platelet (5 fold) and leukocyte (3 fold or more) counts when compared to whole blood. When PC are activated by adding thrombin or a calcium salt, they form a fibrin polymer and are known as platelet gels (PG). Thus, PG from P-PRP is denominated as pure-platelet rich gel (P-PRG) and PG from L-PRP is termed leukocyte-platelet rich gel (L-PRG) [7].

Platelet gels release several growth factors (GF), such as transforming growth factor beta 1 (TGF-β_1_) [8,9] and platelet derived growth factor type BB (PDGF-BB) [10]. These proteins have anti-inflammatory, anabolic and angiogenic effects [11]. Both GF are mainly stored in platelet alpha granules and their release correlates with the degree of platelet activation. The GF profile released from P-PRP or L-PRP will be determined by the cellular components concentrated in each substance [12].

Many questions emerge when a particular PC (either P-PRP or L-PRP) is proposed for regenerative therapy in horses. First, it is essential to know the cellular and GF profile of this substance before its (experimental or clinical) use. Several intrinsic and extrinsic aspects could influence the cellular and molecular features of a PC. Intrinsic factors such as breed, gender and, age or, extrinsic factors associated to the technique used for the PC preparation and activation could potentially influence on the final composition of these substances.

This study evaluates the effect of intrinsic factors such as, the breed, gender and age on the cellular characteristics and release of TGF-β_1_ and PDGF-BB from equine P-PRP/P-PRG. The objectives of this study were: 1) to compare the cellular composition of P-PRP with whole blood and platelet poor plasma (PPP); 2) to compare the concentration of transforming GF beta 1 (TGF-β_1_) and platelet derived GF isoform BB (PDGF-BB) between P-PRP treated with non-ionic detergent (P-PRP+NID), P-PRG (activated with calcium gluconate -CG-), PPP+NID, PPP gel (PPG), and plasma and; 3) to evaluate and to correlate the effect of the breed, gender and age on the cellular and GF concentration for each blood component.

The hypothesis from our study was that intrinsic factors, such as breed, gender and age could influence the final concentration of cells and growth factors in equine P-PRP/P-PRG.

## Results

### General hematological results

**Figure 1:**
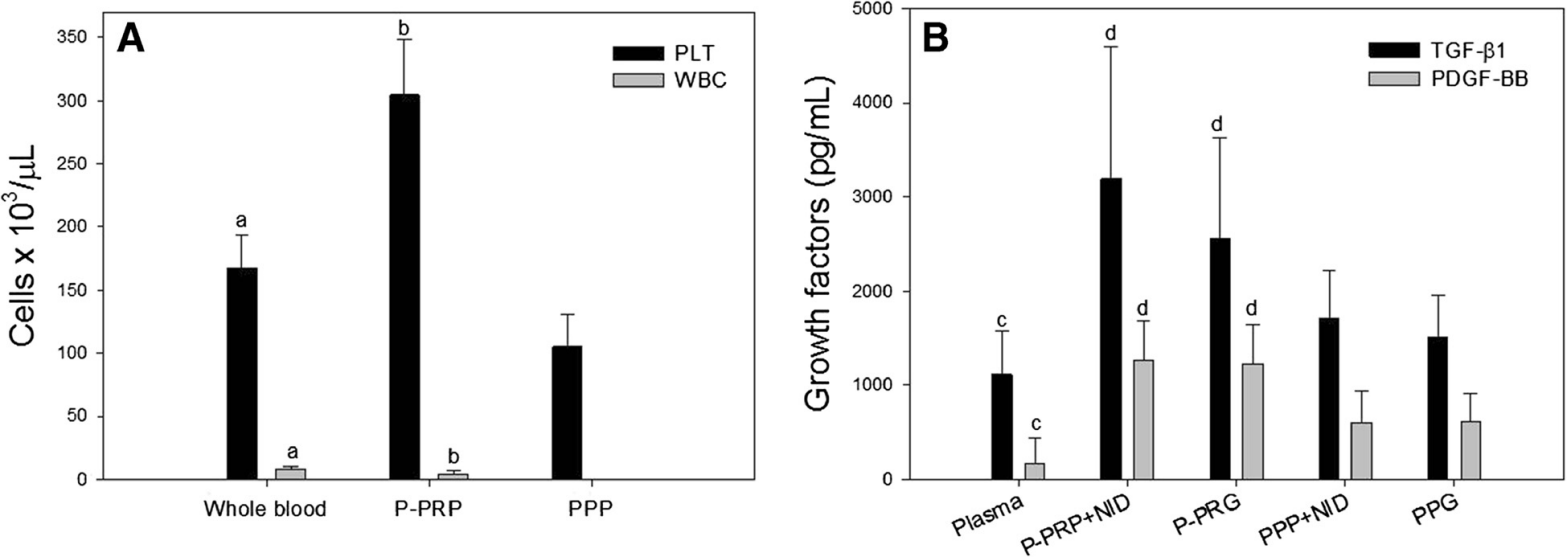
**A) Platelet (PLT) and leukocyte (WBC) concentration in whole blood, P-PRP and, PPP. B**) Transforming growth factor beta 1 (TGF-β_1_) concentrations (pg/mL) in different plasma, P-PRP plus non ionic detergent (P-PRP+NID), pure-platelet rich gel (P-PRG), PPP+NID and platelet poor gel (PPG) Bars represent the mean of n= 40±s.d. Different letters indicate significant differences between groups.

There were statistically significant (P < 0.001) differences for all the general hematological variables evaluated between whole blood, P-PRP and PPP (Figure 1A), except MPV and PDW. Mean platelet volume values were similar between whole blood and PPP and both parameters differed (P < 0.001) from P-PRP. Platelet distribution width values were similar for whole blood and P-PRP; however these PDW values differed (P < 0.001) from same parameter in PPP (Table 1).

**Table 1:** General results for the hematological variables of each blood component

**Variable**	**Whole blood*(n=40)***	**P-PRP*(n=40)***	**PPP*(n=40)***
RBC (10^6^/μL)	6.4 (0.7)^a^	0.14 (0.1)^b^	0.00 (0.0)
WBC (10^3^/μL)	8.2 (1.6)^a^	4.3 (2.2)^b^	0.06 (0.0)
LY (%)	37.0 (8.6)^a^	58.8 (9.5)^b^	- -
MO (%)*	1.8 (0.8)^a^	0.0 (1.2)^b^	- -
EOS (%)*	3.5 (5.0)^a^	0.0 (3.9)^b^	- -
GRA (%)	57.9 (9.7)^a^	38.4 (10.7)^b^	- -
PLT (10^3^/μL)	167.1 (25.9)^a^	304.3 (43.9)^b^	104.9 (25.4)
MPV (fL)	3.4 (0.4)	3.6 (1.0)^a^	3.3 (0.4)
PDW (%)	16.8 (0.7)^b^	17.1 (0.5)^b^	18.4 (0.7)

Data are presented as mean (±s.d.).

P-PRP: Pure-platelet rich plasma. PPP: Platelet poor plasma. RCB: Red blood cells. WBC: White blood cells (leukocytes). LY: Lymphocytes. MO: Monocytes. EOS: Eosinophils. GRA: Granulocytes (neutrophils and basophils). PLT: Platelets. MPV: Mean platelet volume. PDW: Platelet distribution width. a: Significantly different (SD) (P<0.001) from P-PRP and PPP. b: SD (P<0.001) from PPP. *Data are presented as median (interquartile range -IR-).

### General results for total protein, transforming growth factor beta 1 and platelet derived growth factor isoform BB

Total protein concentration was significantly (P < 0.001) higher in P-PRP+NID when compared to plasma, P-PRG, PPP+NID and, PPG. Both TGF-β_1_ and PDGF-BB concentrations (either in pg/mL or pg/mg of TP) were significantly lower for plasma in comparison with the other blood components. P-PRP+NID and P-PRG showed the highest significant concentrations for these GF in comparison with plasma, PPP+NID and, PPG (Table 2 and Figure 1B).

**Table 2:** General results for total protein, transforming growth factor beta 1 and platelet derived growth factor isoform BB for each blood component

**Variable**	**Blood component**				
	**Plasma**	**P-PRP+NID**	**P-PRG**	**PPP+NID**	**PPG**
	***(n=40)***	***(n=40)***	***(n=40)***	***(n=40)***	***(n=40)***
Total protein -TP- (mg/mL)*	45.9 (4.4)^a^	49.5 (6.2)^b^	46.1 (4.5)	46.4 (5.0)	45.5 (5.5)
TGF-β1 (pg/mL)	1114.3 (457.7)^c^	3192.7 (1395.3)^d^	2555.5 (1069.4)^d^	1714.3 (496.3)	1508.0 (445.9)
TGF-β1 (pg/mg of TP)	24.4 (11.2)^c^	64.9 (29.5)^d^	56.0 (24.3)^d^	37.2 (11.2)	33.3 (10.5)
PDGF-BB (pg/mL)	158.1 (281.0)^c^	1259.7 (418.6)^d^	1228.8 (418.0)^d^	601.8 (339.3)	611.7 (295.9)
PDGF-BB (pg/mg of TP)	3.5 (6.5)^c^	25.5 (8.7)^d^	27.2 (10.8)^d^	13.2 (7.9)	13.6 (7.3)

Data are presented as mean (±s.d.).

P-PRG: Pure-platelet rich gel. PPG: Platelet poor gel. NID: Non ionic detergent. TGF-β_1_: Transforming growth factor beta 1. PDGF-BB: Platelet derived growth factor isoform BB. Other initials like in Table 1. a: SD (P<0.001) from P-PRP+NID. b: SD (P<0.001) from P-PRG, PPP+NID and, PPG. c: SD (P<0.001) con P-PRP+NID, P-PRG, P-PPP+NID and PPG. d: SD (P<0.001) from PPP+NID and PPG. *Data are presented as median (IR).

### Breed’s effect on hematological results

Platelet and WBC counts for whole blood and PPP were statistically (P < 0.001) higher for CCH in comparison with ACH. Lymphocyte relative count was significantly (P < 0.001) higher in whole blood from CCH in comparison with ACH. GRA% was significantly (P < 0.001) higher in whole blood and P-PRP from ACH in comparison with CCH. MPV values were significantly lower in PRP for CCH in comparison with ACH and, PDW values were significantly lower (P < 0.001) in PPP from CCH. Pure-platelet rich plasma from CCH presented a significant (P < 0.001) increase of RBC in comparison with the same blood component in ACH (Table 3).

**Table 3:** Hematological results for each breed and their respective blood components

**Variable**	**Argentinean creole horse**			**Colombian creole horse**		
	***(n=20)***			***(n=20)***		
	**Whole blood**	**P-PRP**	**PPP**	**Whole blood**	**P-PRP**	**PPP**
RBC (10^6^/μL)*	6.6 (0.6)^a^	0.0 (0.0)^b^†	0.0 (0.0)	6.2 (1.0)^a^	0.1 (0.2)^b^	0.0 (0.0)
WBC (10^3^/μL)	7.4 (1.2)^a^†	4.1 (1.7)^b^	0.0 (0.0)	9.0 (1.6)^a^	4.4 (2.7)^b^	0.0 (0.0)
LY (%)	33.8 (8.9)^a^‡	55.8 (8.2)^b^	- -	40.2 (7.2)^a^	61.7 (10.1)^b^	- -
MO (%)*	1.7 (0.8)	0.0 (0.4)	- -	1.8 (1.2)^b,e^	0.0 (1.5)^b^	- -
EOS (%)*	2.7 (3.9)^a^	0.0 (2.8)^c^	- -	4.3 (5.6)^b^	0.0 (6.2)^b^	- -
GRA (%)	62.4 (8.5)^a^‡	42.4 (8.7)b‡	- -	53.4 (8.9)^a^	34.3 (11.1)^b^	- -
PLT (10^3^/μL)	154.6 (23.5)^a^†	297.8 (39.1)^b^	96.2 (21.7)‡	179.6 (22.2)^a^	310.8 (48.3)^b^	113.6 (26.4)
MPV (fL)	3.5 (0.4)^d^	3.8 (1.4)b†	3.5 (0.4)‡	3.3 (0.3)	3.5 (0.3)^c^	3.2 (0.2)
PDW (%)*	17.0 (1.1)^b^	17.2 (0.3)b‡	18.5 (1.0)	16.7 (0.9)^b^	16.8 (0.6)^b^	18.2 (1.0)

Data are presented as mean (±s.d.).

The initials like in Table 1. Letters denote SD for each independent breed in each blood component. a: SD (P<0.001) from P-PRP and, PPP. b: SD (P<0.001) from PPP. c: SD (P<0.05) from PPP. d: SD (P<0.001) from P-PRP. e: SD (P<0.05) from P-PRP. †: variable SD (P<0.001) between the two breeds. ‡: variable SD (P<0.05) between the two breeds. *Data are presented as median (IR).

### Breed’s effect on total protein, transforming growth factor beta 1 and platelet derived growth factor isoform BB concentrations

Total protein concentrations were significantly higher (P < 0.001) in P-PRP+NID for both breeds when compared with the other blood components. In ACH, TGF-β_1_ and PDGF-BB (either pg/mL or pg/mg of TP) concentrations were statistically (P < 0.001) higher for P-PRP+NID and P-PRG when compared with the other blood components. The same statistical behavior was noticed for TGF-β_1_ and PDGF-BB concentrations in CCH (Table 4). When both breeds were compared, no differences were found for TGF-β_1_ concentrations between each blood component. However, the PDGF-BB concentrations (either pg/mL or pg/mg of TP) in CCH blood components were significantly higher respect to ACH (Table 4).

**Table 4:** Results for total protein, transforming growth factor beta 1 and platelet derived growth factor isoform BB for each breed and their respective blood components

**Variable**	**Blood component**				
	**Plasma**	**P-PRP+NID**	**P-PRG**	**PPP+NID**	**PPG**
**Argentinean Creole Horse*(n=20)***					
Total protein -TP- (mg/mL)	47.1 (4.6)	49.4 (6.6)^a^	46.2 (4.0)	46.7 (3.4)	46.8 (5.6)†
TGF-β_1_ (pg/mL)	998.0 (521.0)^b^	2988.0 (1192.0)^c^	2644.0 (502.0)^c^	1508.0 (813.0)	1478.0 (876.0)
TGF-β_1_ (pg/mg of TP)	20.7 (10.5)^d,e^	60.0 (30.7)^c^	51.9 (14.8)^c^	31.2 (20.3)	32.0 (20.3)
PDGF-BB (pg/mL)*	69.9 (50.7)^b^†	1060.5 (279.0)^c^‡	978.1 (284.7)^c^§	428.7 (135.2) §	418.2 (103.0)§
PDGF-BB (pg/mg of TP)*	1.5 (1.1)^a^†	22.0 (7.2)^c^‡	21.1 (6.9)^c^§	9.2 (3.6)§	8.8 (2.5)§
**Colombian Creole Horse*(n=20)***					
TP (mg/mL)	45.7 (5.8)^f^	49.6 (5.9)^g,h^	45.9 (5.6)	45.7 (4.7)	44.3 (4.6)
TGF-β_1_ (pg/mL)	1128.0 (383.0)^d,e^	2700.0 (2561.0)^g,h^	1834.0 (2220.0)^d^	1704.0 (884.0)	1456.0 (438.0)
TGF-β_1_ (pg/mg of TP)	24.5 (8.9)^d,e^	58.2 (42.5)^d,g^	42.2 (49.8)^d^	37.1 (16.7)	34.4 (10.9)
PDGF-BB (pg/mL)*	246.3 (378.4)^b^	1458.9 (445.3)^c^	1479.6 (381.1)^c^	775.0 (393.6)	805.2 (300.6)
PDGF-BB (pg/mg of TP)*	5.6 (8.8)^b^	29.0 (8.8)^c^	33.4 (10.6)^c^	17.1 (9.1)	18.4 (7.4)

Data are presented as median (interquartile range).

Initials like in Table 2. Letters denote significant differences (SD) for each independent breed and their respective blood component. a: SD (P<0.05) from PRG. b: SD (P<0.003) from PRP+NID, P-PRG, PPP+NID and, PPG. c: SD (P<0.001) from PPP+NID and, PPG. d: SD (P<0.05) from PPG. e: SD (P<0.001) from PRP+NID, P-PRG and, PPP+NID. f: SD (P<0.05) from PRP+NID. g: SD (P<0.05) from PPP+NID. h: SD (P<0.001) from PPG. †: SD (P<0.05) between breeds. ‡: SD (P<0.009) between breeds. §: SD (P<0.001) between breeds. *Data are presented as mean (±s.d).

### Gender’s effect on hematological results

Cellular counts, MPV and, PDW values for whole blood, P-PRP and, PPP for each independent gender presented a similar statistical behavior than the hematological general results. However, PLT counts for whole blood, P-PRP and, PPP were significantly (P < 0.001) higher in females in comparison with males (Table 5).

**Table 5:** Hematological results for gender and their respective blood components

**Variable**	**Male**			**Female**		
	***(n=30)***			***(n=10)***		
	**Whole blood**	**P-PRP**	**PPP**	**Whole blood**	**P-PRP**	**PPP**
RBC (10^6^/μL)	6.5 (0.7)^a^	0.1 (0.1)^b^	0.0 (0.0)	6.2 (0.9)^a^	0.1 (0.1)^b^	0.0 (0.0)
WBC (10^3^/μL)	8.0 (1.5)^a^	4.0 (1.8)^b^	0.0 (0.0)	8.8 (1.7)^b,c^	5.2 (3.1)^b^	0.0 (0.0)
LY (%)	35.9 (8.8)^a^	58.2 (9.9)^b^	- -	40.5 (7.2)^a^	60.6 (8.6)^b^	- -
MO (%)*	1.8 (0.8)	0.0 (0.9)	- -	1.8 (1.5)^b^	0.0 (1.6)^d^	- -
EOS (%)*	3.1 (3.8)	0.0 (4.0)	- -	5.1 (5.2)^b^	0.0 (3.8)^d^	- -
GRA (%)	59.6 (9.4)^a^	39.2 (10.4)^b^	- -	52.7 (9.0)^b,c^	35.8 (11.7)^b^	- -
PLT (10^3^/μL)	159.7 (22.7)^a^†	295.7 (44.2)^b^‡	99.8 (23.3)‡	189.1 (22.7)^a^	329.9 (32.7)^b^	120.0 (26.8)
MPV (fL)	3.4 (0.4)^c^	3.6 (1.1)b	3.4 (0.4)	3.4 (0.3)	3.7 (0.2)^d^	3.2 (0.2)
PDW (%)	16.9 (0.7)^b^	17.1 (0.5)^b^	18.5 (0.7)	16.6 (0.8)^b^	17.0 (0.5)^b^	18.2 (0.8)

Data are presented as mean (±s.d.).

The initials like in Table 1. Letters denote SD for each independent sex in each blood component. a: SD (P<0.001) from P-PRP and, PPP. b: SD (P<0.001) from PPP. c: SD (P<0.05) from P-PRP. d: SD (P<0.05) from PPP. †: variable SD (P<0.001) between males and females. ‡: variable SD (P<0.05) between males and females. *Data are presented as median (IR).

### Gender’s effect on total protein, transforming growth factor beta 1 and platelet derived growth factor isoform BB concentrations

Total protein, TGF-β_1_ and PDGF-BB (either in pg/mL or pg/mg of TP) concentrations for each blood component for each sex presented a same statistical behavior than for the general results (Table 6). However, when genders were compared, females presented significantly higher plasma TGF-β_1_ (pg/mg of TP) concentrations than males. PDGF-BB concentrations for P-PRP+NID, P-PRG, PPP+NID and, PPG were significantly higher in females in comparison with males (Table 6).

**Table 6:** Results for total protein, transforming growth factor beta 1 and platelet derived growth factor isoform BB for gender and their respective blood components

**Variable**	**Blood component**				
	**Plasma**	**P-PRP+NID**	**P-PRG**	**PPP+NID**	**PPG**
**Males*(n=30)***					
TP (mg/mL)*	47.1 (4.6)	49.4 (6.6)^a^	46.2 (4.0)†	46.7 (3.4)	46.8 (5.6)
TGF-β_1_ (pg/mL)	1019.0 (467.8)^b,c^	3325.8 (1108.1)^d^	2536.6 (582.3)^d^	1674.8 (533.3)	1512.6 (549.7)
TGF-β_1_ (pg/mg of TP)	21.9 (11.6)^b,c^†	69.4 (28.2)^d^	54.9 (14.8)^d^	35.6 (11.1)	32.5 (13.4)
PDGF-BB (pg/mL)	69.9 (50.7)^e^	1060.5 (279.0)^d^†	978.1 (284.7)^d^‡	428.7 (135.2)§	418.2 (103.0)‡
PDGF-BB (pg/mg of TP)	1.5 (1.1)^e^	22.0 (7.2)^d^†	21.1 (6.9)^d^‡	9.2 (3.6)§	8.8 (2.5)‡
**Females*(n=10)***					
TP (mg/mL)*	45.0 (5.1)	49.5 (7.8)	43.5 (6.7)	44.3 (5.1)	42.8 (4.7)
TGF-β_1_ (pg/mL)	1185.6 (508.9)^f^	3608.0 (1906.2)^c^	2892.8 (1749.0)	1704.8 (504.4)	1410.0 (197.4)
TGF-β_1_ (pg/mg of TP)	26.7 (12.5)^f^	71.2 (34.8)^c^	64.9 (37.1)	37.9 (11.3)	32.8 (4.9)
PDGF-BB (pg/mL)	333.6 (477.8)^g^	1535.5 (444.6)^h^	1589.1 (363.7)^h^	864.0 (463.0)	933.7 (319.9)
PDGF-BB (pg/mg of TP)	7.7 (11.2)^i^	30.6 (8.0)	36.8 (10.3)^h^	19.4 (10.8)	21.7 (7.7)

Data are presented as mean (±s.d.).

The initials like in Table 2. Letters denote SD for each independent sex and their respective blood components. a: SD (P<0.05) from P-PRG. b: SD (P<0.001) from P-PRP+NID, P-PRG and, PPP+NID. c: SD (P<0.05) from PPG. d: SD (P<0.001) from PPP+NID and, PPG. e: SD (P<0.001) from P-PRP+NID, P-PRG, PPP+NID and, PPG. f: SD (P<0.05) from P-PRP+NID. g: SD (P<0.001) from P-PRP+NID and, P-PRG. h: SD (P<0.05) from PPP+NID and, PPG. i: SD (P<0.05) from P-PRP+NID, P-PRG, PPP+NID and, PPG. †: variable SD (P<0.05) between males and females. ‡: variable SD (P<0.001) between males and females. §: variable SD (P<0.003) between males and females. *Data are presented as median (IR).

### Age’s effect on hematological results

Cellular counts, MPV and, PDW values for whole blood, P-PRP and, PPP for each independent age group presented a similar statistical behavior than for the hematological general results. Horses younger than 5 years old presented significant higher LY% and lower GRA% than the age group of horses between 5.1-10 years old and the group of horses older than 10.1 years old (Table 7).

**Table 7:** Hematological results for age groups and their respective blood components

**Variable**	**Horses younger than 5 years old**			**Horses between 5.1-10 years old**			**Horses older than 10.1 years old**		
	***(n=7)***			***(n=20)***			***(n=13)***		
	**Whole blood**	**P-PRP**	**PPP**	**Whole blood**	**P-PRP**	**PPP**	**Whole blood**	**P-PRP**	**PPP**
RBC (10^6^/μL)	6.8 (1.1)^a^	0.1 (0.1)^b^	0.0 (0.0)	6.4 (0.5)^a^	0.1 (0.0)^c^	0.0 (0.0)	6.3 (0.8)^a^	0.1 (0.2)^c^	0.0 (0.0)
WBC (10^3^/μL)*	8.0 (2.9)^c^	6.0 (2.4)^c^	0.0 (0.1)	7.5 (2.2)^a^	4.3 (2.1)^c^	0.0 (0.0)	8.3 (1.9)^a^	3.3 (1.5)^c^	0.1 (0.0)
LY (%)	43.6 (5.6)^a^†	61.0 (7.5)^c^	- -	38.9 (7.8)^a^†	61.1 (9.1)^c^	- -	30.5 (7.3)^a^	54.0 (10.0)^c^	- -
MO (%)*	1.2 (1.5)	0.0 (0.0)	- -	1.8 (1.3)^a^	0.0 (1.3)^b^	- -	1.9 (1.1)	0.0 (1.3)	- -
EOS (%)*	4.9 (7.2)^d^	0.0 (0.0)	- -	4.1 (5.4)	0.0 (3.1)	- -	2.5 (2.8)	0.0 (4.4)	- -
GRA (%)	49.9 (4.6)^a^†	36.3 (4.4)^c^	- -	56.6 (8.8)^a^†	36.1 (11.3)^c^	- -	64.2 (9.5)^a^	42.9 (11.1)^c^	- -
PLT (10^3^/μL)	170.5 (21.2)^b,e^	327.0 (50.5)^b^	110.4 (32.3)	165.7 (31.2)^a^	304.5 (48.1)^c^	103.2 (26.3)	167.4 (19.9)^a^	291.6 (29.1)^c^	104.5 (21.4)
MPV (fL)	3.2 (0.3)	3.0 (1.3)	3.1 (0.3)	3.5 (0.4)	3.8 (1.1)	3.4 (0.4)	3.4 (0.2)^f^	3.8 (0.3)^b^	3.3 (0.3)
PDW (%)	16.6 (1.0)^b^	16.7 (0.4)^b^	18.6 (0.9)	16.6 (0.7)^a^	17.1 (0.5)^c^	18.2 (0.6)	17.2 (0.6)^c^	17.3 (0.6)^c^	18.7 (0.7)

Data are presented as mean (±s.d.).

The initials like in Table 1. Letters denote SD for each independent age group in each blood component. a: SD (P<0.001) from P-PRP and, PPP. b: SD (P<0.05) from PPP. c: SD (P<0.001) from PPP. d: SD (P<0.05) from P-PRP and, PPP. e: SD (P<0.001) from P-PRP. f: SD (P<0.05) from P-PRP. †: SD (P<0.05) from horses older than 10.1 years old.*Data are presented as median (IR).

### Age’s effect on total protein, transforming growth factor beta 1 and platelet derived growth factor isoform BB concentrations

Total protein, TGF-β_1_ and, PDGF-BB concentrations for each blood component for each independent age group presented the same trend in the statistical behavior than for the general results (Table 8). Horses younger than 5 years old presented significant lower TP concentrations than the other age groups. The same group of horses presented significant (P < 0.001) higher concentrations of PDGF-BB in P-PRP+NID when compared with the age group of horses between 5.1-10 years old and the group of horses older than 10.1 years old. In addition, P-PRG from the group of horses younger than 5 years old presented higher concentrations of PDGF-BB (pg/mg of TP) in comparison with the other age groups (Table 8).

**Table 8:** Results for total protein, transforming growth factor beta 1 and platelet derived growth factor isoform BB for age groups and their respective blood components

**Variable**	**Blood component**				
	**Plasma**	**P-PRP+NID**	**P-PRG**	**PPP+NID**	**PPG**
**Horses younger than 5 years old*(n=7)***					
TP (mg/mL)	42.0 (3.6)†	48.2 (7.1)	43.2 (5.3)	42.7 (5.5)†	41.4 (4.7)
TGF-β_1_ (pg/mL)	1124.0 (216.0)^a^	3584.0 (2044.0)^b^	3284.0 (2176.0)	1732.0 (844.0)	1456.0 (336.0)
TGF-β_1_ (pg/mg of TP)	26.8 (6.4)^c^	74.2 (42.6)^d^	72.4 (50.6)^e^	41.0 (16.8)	37.1 (6.9)
PDGF-BB (pg/mL)	106.7 (165.8)^f,g^	1417.0 (840.0)^e^†	1585.0 (992.7)	494.9 (864.0)	677.6 (810.6)
PDGF-BB (pg/mg of TP)	2.4 (4.1)^h,i^	33.2 (12.1)†	34.5 (29.0)†	12.3 (21.3)	15.1 (20.1)
**Horses between 5.1-10 years old*(n=20)***					
PT (mg/mL)	47.1 (3.9)^j^	50.0 (6.0)^e,i^	46.4 (3.9)	47.2 (5.8)	45.9 (5.6)
TGF-β_1_ (pg/mL)	1054.0 (633.0)^f,k^	2890.0 (2048.0)^b^	2366.0 (1044.0)^k,l^	1560.0 (797.0)	1456.0 (593.0)
TGF-β_1_ (pg/mg of TP)	22.4 (14.5)^f,k^	56.7 (40.4)^b^	49.2 (31.5)^k,l^	31.3 (16.6)	30.5 (15.7)
PDGF-BB (pg/mL)	72.1 (77.4)^m^	1180.5 (810.9)^b^	1140.5 (599.8)^b^	454.8 (353.3)	513.9 (301.9)
PDGF-BB (pg/mg of TP)	1.5 (1.3)^m^	25.0 (14.1)^b^	24.4 (12.9)^b^	9.8 (9.9)	11.1 (6.8)
**Horses older than 10.1 years old*(n=13)***					
TP (mg/mL)	46.7 (4.7)	49.1 (8.2)	46.9 (4.2)	46.8 (2.2)	45.8 (3.4)
TGF-β_1_ (pg/mL)	1012.0 (426.0)^d,f^	2696.0 (1678.0)^b,e^	2264.0 (1196.0)	1592.0 (896.0)	1520.0 (806.0)
TGF-β_1_ (pg/mg of TP)	22.2 (8.9)^e,n^	55.9 (23.4)	48.4 (16.6)	33.9 (17.6)	35.1 (11.4)
PDGF-BB (pg/mL)	59.3 (131.7)^m^	1007.0 (630.6)^b^	1208.0 (624.3)^b^	523.6 (207.6)	562.5 (268.6)
PDGF-BB (pg/mg of TP)	1.2 (3.0)^m^	21.6 (13.1)^l,o^	25.7 (16.4)^b^	10.4 (5.5)	12.8 (6.2)

Data are presented as mean (±s.d.).

The initials like in Table 2. Letters denote SD for each independent age group and their respective blood components. a: SD (P<0.05) from P-PRP+NID and, P-PRG. b: SD (P<0.001) from PPP+NID and, PPG. c: SD (P<0.05) from the all blood components. d: SD (P<0.05) from PPP+NID and, PPG. e: SD (P<0.001) from PPG. f: SD (P<0.001) P-PRP+NID and, P-PRG. g: SD (P<0.003) from PPP+NID and, PPG. h: SD (P<0.001) from PRP+NID. i: SD (P<0.05) from P-PRG. j: SD (P<0.05) from P-PRP+NID. k: SD (P<0.05) from PPP+NID. l: SD (P<0.001) from PPG. m: SD (P<0.001) from the all blood components. n: SD (P<0.001) from P-PRP+NID, P-PRG and, PPP+NID. o: SD (P<0.001) from PPP+NID. †: variable SD (P<0.05) from horses between 5.-10 years old and horses older than 10.1 years old.

### General correlations

Significant (P < 0.001) positive correlations were detected between PLT and PDGF-BB concentration (*r*_*s*_*=* 0.80), PLT and TGF-β_1_ concentration (*r*_*s*_*=* 0.60), WBC count and PDGF-BB concentration (*r*_*s*_*=* 0.66) and, TGF-β_1_ and PDGF-BB concentration (*r*_*s*_*=* 0.67). A significant (P < 0.001) negative correlation was observed between PDW% and PDGF-BB concentration (*r*_*s*_*= −*0.71).

### Specific correlations for each blood component and the gender sex and, age

Significant (P < 0.001) positive correlations were observed between breed and PDGF-BB concentrations from P-PRG (*r*_*s*_*=* 0.62), breed and PDGF-BB concentrations from PPG (*r*_*s*_*=* 0.79).

## Discussion and conclusion

Results from this study demonstrate that the double centrifugation tube method is a reliable technique for producing equine P-PRP, such as it was demonstrated previously [8]. This method was initially evaluated in a mixed group of 26 horses of different breeds, ages and, gender [8]. In that study PLT concentration was lower and WBC concentration was higher in P-PRP [8] in comparison with the hematological results from our study. However, it was no possible to find significant differences associated with the intrinsic variables [8] such as was found in the present research. Thus, results from our study (using a larger sample size of horses) suggest that centrifugation protocols for producing equine P-PRP and perhaps L-PRP, should be adapted in function of the breed, since possibly the size and weight of PLT and WBC could be different for each equine specific breed.

Some studies suggest that, cellular content will influence the GF concentration in a determined PC [12]. This fact was evidenced when TGF-β_1_ and PDGF-BB concentrations were measured in the different blood components of our study. However, although there are several equine studies evaluating the GF concentration in PRP, there is conflicting results in the TGF-β_1_ and PDGF-BB concentrations from equine PG obtained by either manual methods [8,13] or semi-automated devices [9,10,14,15]. The technique used for GF determination and non-standardized technical aspects inherent to each research, prevent comparing our findings with some of these studies.

However, our GF results could be compared with the TGF-β_1_ and PDGF-BB concentrations reported by Textor *et al.*[10] for equine PRP produced by a manual method and a semi-automated device. They reported the concentration for these GF in PRP+NID and compared their release from PRP activated with two collagen type I (COL1) concentrations, 10 and 20 μg/mL [10]. In our study, lower TGF-β_1_ and PDGF-BB concentrations (mean ± d.s., 3192.7 ± 1395.3 and 1259.7 ± 418.6 pg/mL, respectively), were obtained from P-PRP+NID in comparison with equine PRP+NID (22677 ± 12125 and 4332 ± 2212 pg/mL, respectively) reported by Textor *et al.*[10]. The difference observed for GF concentrations in these blood components could be associated with a higher number of PLT and WBC concentrated in PRP+NID from Textor *et al.*’s study [10]. On the other hand, PRP activated with COL1 produced a very lower GF release in comparison with the TGF-β_1_ and PDGF-BB concentration released from P-PRG activated with CG at 6 h. The findings of our study suggest that CG produces a complete release of GF from P-PRG in the first 6 h post-activation. In contrast, COL1 produced a very weak GF release from PRP [10].

Our study demonstrated that intrinsic factors such as, breed, gender and age influence the cellular composition of P-PRP and GF content in P-PRG. These findings are novel and should be considered in the clinical use of these bioproducts. However, it is important to consider that these observations should also be confirmed for either equine L-PRP or L-PRG obtained with semi-automated devices using an adequate simple size of horses to avoid unpowered studies with erroneous conclusions [16].

The most intriguing result of our study was the significantly higher PDGF concentrations in P-PRP+NID and P-PRG derived from CCH in comparison with ACH. This same aspect was also observed in females in comparison with males and in horses younger than 5 years old in comparison with older horses. To note, CCH is a pony-like breed and our findings could suggest that PDGF-BB from platelets could be a pivotal factor associated with accelerated limb wound healing in ponies in comparison with horses [17]. It is important to bear in mind that anabolic GF, such as insulin like growth factor type I is also highly expressed and produced in young horses in comparison with older ones [18]. The main limitation of our study was associated with the fact that only CCH females were used for gender comparison. However, this is the first time that significant differences between genders for PLT count in P-PRP and PDGF-BB concentrations in P-PRG have been described in horses. At least, two aspects could be useful to explain these differences. First, the present study used a sample size greater than in other equine studies [3,8-10,13]. This situation added statistical power to the study and increased the possibility of finding significant differences for the evaluated variables [16]. Second, gender differences have been reported for PLT and WBC counts in human beings with greater concentration of these cells in females [19]. While it is known that hormonal influences could be associated with these hematological gender differences in human beings [20], we did not measure the concentration of sexual hormones in horses from our study to support this hypothesis.

The correlation analysis from our study suggests that PDGF-BB is one of the main GF contained in equine PLT. On the other hand, TGF-β_1_concentrations were associated with both PLT and WBC counts. Of note, PDGF-BB concentrations were correlated with the type of breed, gender and the age. These findings suggest that this GF should be used as an indicator of PLT enrichment in PRP and as indicator of equine PRG quality.

Taken together, our data suggests that intrinsic factors, such as breed, sex and age can influence the cellular and GF profile of equine P-PRP/P-PRG. Specific protocols for concentrating platelets and GF by using manual or semi-automated devices should be standardized in function of the equine intrinsic factors. Additional studies are necessary to know if these intrinsic factors could influence the therapeutic potential of P-PRP/P-PRG in horses.

## Methods

This study was approved by the Ethical Committee of the Universidad de Caldas.

### Horses

Forty clinically normal horses of two breeds were used. 20 animals were Argentinean Creole Horses (ACH) and, 20 were Colombian Creole Horses (CCH). The ACH were geldings with a mean age of 10.7 (± 6.2) years old. Of the CCH, 10 were mares and, 10 were geldings. These horses had a mean age of 8.5 (± 4.1) years old.

### Blood collection and preparation of the platelet concentrates

**Figure 2:**
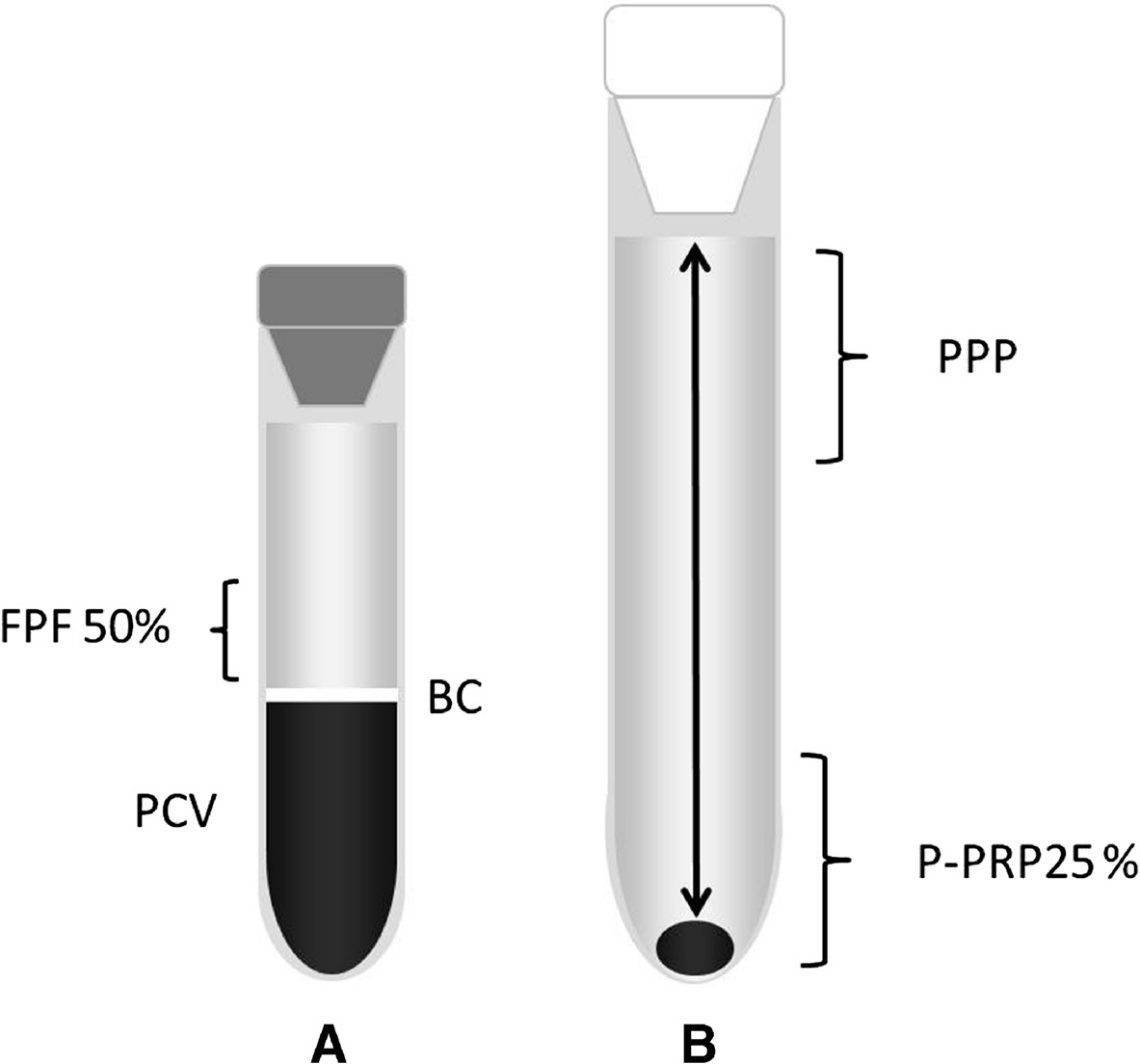
**Graphic illustrating the plasma fractions obtained from the tube-method protocol.** Left tube **(A)** contains the first plasma fraction (50%) (FPF) obtained by the single centrifugation tube method. Right tube **(B)** contains pure-platelet rich plasma (P-PRP) and platelet poor plasma (PPP). BC: Buffy coat. PCV: packed cell volume.

Blood was drawn from jugular venipuncture and deposited in 8.5 mL tubes with ACD solution A (ACD-A) (BD Vacutainer®, Becton Drive, Franklin Lakes, NJ, USA). After centrifugation at 120 *g* for five minutes, the first 50% of the top supernatant plasma fraction, adjacent to the buffy coat, was collected. This fraction was then centrifuged at 240 *g* for five minutes and the bottom fourth fraction was collected [8]. This fraction was considered P-PRP. The upper plasma fraction P-PRP was considered as PPP (Figure 2). Plasma was obtained by centrifugation of ACD-A blood at 5500 *g* for 8 min.

### Haematological analysis

A complete automated hemogram (Celltac-α MEK 6450, Nihon Kodhen, Japan) was performed in duplicate for whole blood, P-PRP and, PPP. Platelet (PLT) count, mean platelet volume (MPV fL), platelet distribution width (PDW %), total leukocyte (WBC) count, and relative count of neutrophils and basophils (GRA), lymphocytes (LY), monocytes (MON), eosinophils (EOS) and, packed cell volume (PCV) were determined.

### Activation of platelet concentrates

Six hundred μL of a 10% calcium gluconate (CG) solution (9.3 mg/mL) (Ropsohn Therapeutics Ltda®, Bogotá, Colombia) was added to 6 mL of P-PRP or PPP for producing P-PRG and PPG, respectively. P-PRG and PPG were incubated at 37°C for 6 h to stimulate GF release. Clots were mechanically released from the walls of the tubes and centrifuged at 5500 *g* for 8 min. The resulting supernatant was aliquoted, and frozen at −82°C for later determination of TGF-β_1_ and PDGF-BB.

### Lysis of platelet concentrates

Samples of P-PRP and PPP were incubated at 37°C during 15 min with 600 μL of a solution containing 0.5% of a non-ionic detergent (NID) (Triton®X100, Panreac Química, Barcelona, Spain). Platelet concentrates treated with NID were used as a positive control of GF release [11]. Lysates were processed in a similar fashion than supernatants from P-PRG and PPG.

### Total protein determination

Total protein (TP) concentration from all the samples was determined using the biuret method (Proteína total (Biuret), BioSystems, Barcelona, Spain) [21] followed by spectrophotometric quantification.

### Determination of TGF-β_1_ and PDGF-BB concentration by ELISA

The TGF-β_1_ and PDGF-BB concentrations from supernatants and lysates of each blood component were determined in duplicate by sandwich ELISA developed with commercial antibodies for human TGF-β_1_ (Human TGF-β1, DY240E, R&D Systems, Inc., Minneapolis, USA) [22] and PDGF-BB (Human PDGF-BB, DY220, R&D Systems, Inc., Minneapolis, USA) [23]. Both ELISA were performed according to manufacturers’ instructions. Readings were performed at 450 nm.

### Statistical analysis

Data were analyzed using a commercial software (SPSS 18.0, IBM, Chicago, Illinois, USA). A general analysis for comparing the cell and GF concentration between the different hematological components was performed. The effects of the breed (ACH and CCH), gender (male and female) and age (horses younger than 5 years old, horses between 5.1-10 years old and horses older than 10.1 years old) on the hematological parameters and the TGF-β_1_ and PDGF-BB for the different blood components were analyzed.

When, variables presented normal distribution (Shaphiro-Wilk test, P > 0.05), they were presented as means (**± **s.d.) and evaluated by parametric tests (i.e. t-student test for paired samples, one way ANOVA test and, Games-Howell test (for *post-hoc* paired comparisons). Non parametric variables (Shaphiro-Wilk test, P<0.05) were presented as medians (interquartile range -IR-) and, evaluated by using a Kruskal-Wallis test followed when necessary for a U-Mann–Whitney test. Wilcoxon test was used for non related paired comparisons. All the variables were analyzed for general and specific correlations by using a Spearman (*r*_*s*_) test. A P ≤ 0.05 was asset as significant for all the tests.

## Abbreviations

ACD-A: Acid citrate dextrose solution A; ACH: Argentinean creole horses; BC: Buffy coat; CG: Calcium gluconate; CCH: Colombian creole horses; COL1: Collagen type I; EOS: Eosinophils; FPF: First plasma fraction; GF: Growth factors; WBC: Leukocytes (white blood cells); L-PRP: Leukocyte-platelet rich plasma; LY: Lymphocytes; MPV: Mean platelet volume; MO: Monocytes; GRA: Neutrophils and basophils; NID: Non-ionic detergent; PCV: Packed cell volume; PLT: Platelet; PC: Platelet concentrates; PDGF-BB: Platelet derived growth factor isoform BB; PDW: Platelet distribution width; PG: Platelet gels; PPG: Platelet poor plasma gel; PPP: Platelet poor plasma; PRF: Platelet rich fibrin; P-PRP: Pure-platelet rich plasma; P-PRG: Pure-platelet rich gel; RBC: Red blood cells; TP: Total protein; TGF-β_1_: Transforming growth factor beta 1.

## Competing interests

Authors declare no competing interests related with this manuscript.

## Authors’ contributions

JUC, IJS, MP and CEG conceived of the study. JUC, CEG and CL collected samples. CEG, MEA and CL performed the laboratory tests. CEG and JUC performed the statistical analysis. All the authors participated in the drafting of the manuscript. JUC coordinated the study. All authors read and approved the final manuscript.
